# Real-World Use of Sotrovimab for Pre-Emptive Treatment in High-Risk Hospitalized COVID-19 Patients: An Observational Cross-Sectional Study

**DOI:** 10.3390/antibiotics11030345

**Published:** 2022-03-05

**Authors:** Sean W. X. Ong, Dongdong Ren, Pei Hua Lee, Stephanie Sutjipto, Christopher Dugan, Bo Yan Khoo, Jun Xin Tay, Shawn Vasoo, Barnaby E. Young, David C. Lye

**Affiliations:** 1 National Centre for Infectious Diseases, 16 Jln Tan Tock Seng, Singapore 308442, Singapore; sean.ongwx@mohh.com.sg (S.W.X.O.); dongdong.ren@mohh.com.sg (D.R.); peihua.lee@mohh.com.sg (P.H.L.); stephanie.sutjipto@mohh.com.sg (S.S.); christopher_dugan@ttsh.com.sg (C.D.); boyan.khoo@mohh.com.sg (B.Y.K.); jun_xin_tay@ncid.sg (J.X.T.); shawn_vasoo@ncid.sg (S.V.); barnaby_young@ncid.sg (B.E.Y.); 2 Department of Infectious Diseases, Tan Tock Seng Hospital, Singapore 308433, Singapore; 3 Lee Kong Chian School of Medicine, Nanyang Technological University, Singapore 308232, Singapore; 4 Yong Loo Lin School of Medicine, National University of Singapore, Singapore 117597, Singapore

**Keywords:** COVID-19, SARS-CoV-2, sotrovimab, treatment, monoclonal antibody

## Abstract

Data on use of monoclonal antibodies (mAbs) in hospitalized patients are limited. In this cross-sectional study, we evaluated the use of mAbs for early treatment of unvaccinated hospitalized patients with mild-to-moderate COVID-19. All inpatients at our center were screened on 27 October 2021. Primary outcome was in-hospital deterioration as defined by a composite of oxygen requirement, intensive care unit (ICU) admission, or mortality within 28 days of admission. Ninety-four out of 410 COVID-19 inpatients were included in the final analysis, of whom 19 (20.2%) received early treatment with sotrovimab. The median age was 73 years (IQR 61–83), and 35 (37.2%) were female. Although the treatment group was significantly older and had more comorbidities, there was a lower proportion of progression to oxygen requirement (31.6% vs. 54.7%), ICU admission (10.5% vs. 24.0%), or mortality (5.3% vs. 13.3%). Kaplan–Meier curves showed a significant difference in time to in-hospital deterioration (log-rank test, *p* = 0.043). Cox proportional hazards model for in-hospital deterioration showed that sotrovimab treatment was protective (hazard ratio, 0.41; 95% CI, 0.17–0.99; *p* = 0.047) after adjustment for baseline ISARIC deterioration score. Our findings support the use of sotrovimab for early treatment in hospitalized patients with mild-to-moderate COVID-19 at a high risk of disease progression.

## 1. Introduction

Coronavirus disease 2019 (COVID-19), caused by the severe acute respiratory syndrome coronavirus 2 (SARS-CoV-2), can result in severe pneumonia with respiratory failure. While vaccination is highly protective against severe disease [1], there are many who remain unvaccinated and susceptible. Early therapeutics can reduce the risk of progression to severe disease in these patients. Monoclonal antibodies (mAbs) such as sotrovimab, casirivimab/imdevimab (REGEN-COV), and bamlanivimab/etesevimab have been shown to be effective at reducing the risk of subsequent hospitalization or death [2,3,4] in COVID-19 outpatients and have since received US Food and Drug Administration (FDA) emergency-use authorization (EUA) for this indication.

Data regarding hospitalized patients, however, are limited. A clinical trial evaluating bamlanivimab in hospitalized patients did not demonstrate efficacy and was terminated early [5]. In contrast, the RECOVERY trial found reduced 28-day mortality amongst seronegative patients who received REGEN-COV [6]. Accumulating real-world data will help to identify subgroups who may derive the greatest benefit from mAb therapy.

In Singapore, as part of a more conservative hospital-based care model, COVID-19 patients at high risk of severe disease (based on age, vaccination status, and comorbidities) are hospitalized for inpatient observation rather than managed outpatient. We conducted a single-time-point cross-sectional study at the National Centre for Infectious Diseases (NCID), the largest COVID-19 treatment center locally, to evaluate the use of mAbs amongst such hospitalized patients, and we studied the impact of mAb therapy on disease progression.

## 2. Methods

All patients who were, at time of screening, admitted to the NCID were screened on 27 October 2021, regardless of original admission date. Admission dates of the patients ranged from 26 September to 27 October 2021. Inclusion criteria were confirmed COVID-19 by SARS-CoV-2-specific polymerase chain reaction (PCR), admission within first five days of illness, and absence of oxygen requirement at presentation. Patients who were fully vaccinated (defined as two doses of any COVID-19 vaccine more than two weeks before illness onset) were excluded. For patients asymptomatic at presentation, illness onset was defined as the first positive PCR date. These inclusion criteria were based on the original phase III data evaluating outpatient sotrovimab use, which included only patients with ≤5 days of symptoms [2].

Clinical and demographic data were collected by using a standardized data-collection form by the study team. Primary outcome was in-hospital deterioration as defined by a composite outcome of oxygen requirement, intensive care unit (ICU) admission, or mortality. Outcomes were censored upon discharge or at 28 days of illness. Waiver of retrospective data collection was approved by the institutional ethics committee (National Healthcare Group Domain Specific Review Board reference number 2020/01122).

Baseline characteristics were compared by using Fisher’s exact test for categorical variables and Mann–Whitney U test for continuous variables. Time to in-hospital deterioration was assessed by using Kaplan–Meier curves and compared by using log-rank test. A multivariable Cox proportional hazards model was constructed to assess the impact of sotrovimab treatment after adjustment for the admission ISARIC (International Severe Acute Respiratory and Emerging Infections Consortium) deterioration score. This is a well-validated score incorporating multiple clinical variables (age, sex, comorbidities, presence of chest radiographic infiltrates, Glasgow Coma Scale, respiratory rate, oxygen saturation, urea, C-reactive protein, and lymphocyte count) to predict risk of in-hospital deterioration [7], and it has been validated in the local setting in a cohort of hospitalized patients from NCID [8]. Efron’s method was used to handle tied failures, and the proportional hazards assumption was tested by using Schoenfeld’s residuals. A *p*-value <0.05 was considered significant, and all tests were two-tailed. Data analysis was performed on STATA Version 13.0 (StataCorp, College Station, TX, USA), and plots were graphed by using GraphPad Prism Version 9.2 (GraphPad Software, San Diego, CA, USA).

## 3. Results

**Figure 1:**
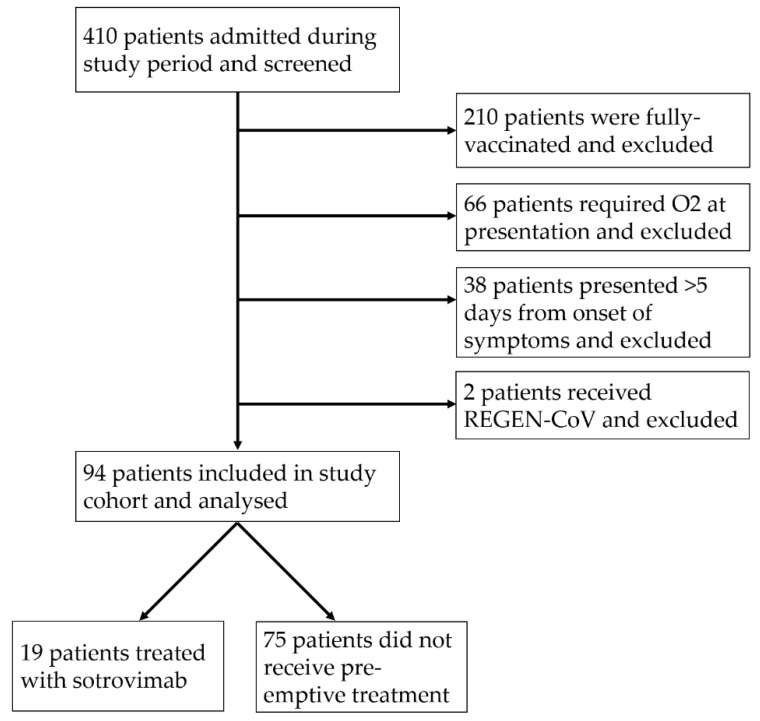
Study flowchart.

A total of 410 patients were admitted on the cross-sectional sampling date and screened, of whom 96 patients met the inclusion and exclusion criteria (study flowchart in Figure 1). Two patients received REGEN-COV and were excluded due to the small number, leaving 94 for the final analysis. Bamlanivimab/etesevimab was not available locally during the study period. Of these 94 patients, 19 (20.2%) were treated with a single dose of sotrovimab after admission. The median age was 73 years (interquartile range (IQR) 61–83), and 35 (37.2%) were female. Table 1 summarizes the baseline characteristics of the study cohort. Of note, the sotrovimab group was significantly older (median age 81 vs. 70 years, *p* = 0.0023) and had more comorbidities (median number of comorbidities three in treatment group vs. two in untreated group). Patients in the sotrovimab treatment group presented to hospital earlier (median day of illness at presentation 2 days vs. 3 days in the untreated group).

Biomarkers of severity, such as white blood cell count, lymphocyte count, C-reactive protein, and lactate dehydrogenase, were not significantly different between the two groups, and there was a similar proportion of patients with pneumonia on baseline chest radiograph (36.8% in treatment vs. 40.0% in control groups). Significant differences in hemoglobin, platelet count, serum creatinine, and urea levels were likely to be reflective of the greater comorbidity burden in the treatment group. However, the median ISARIC mortality score was higher in the sotrovimab treatment group (9 vs. 7), reflecting their older age and increased comorbidity burden.

The sotrovimab treatment group had lower proportions of progression to oxygen requirement (31.6% vs. 54.7%), ICU admission (10.5% vs. 24.0%), or mortality (5.3% vs. 13.3%), though this difference was not statistically significant (Table 1). A greater proportion of patients in the untreated group subsequently received remdesivir (70.7% vs. 36.8%) after deterioration compared to the sotrovimab treatment group, reflective of this greater frequency of severe disease. While a slightly higher proportion of patients in the untreated group subsequently received dexamethasone (54.7% vs. 31.6%), this difference was not statistically significant, likely due to the small sample size. There was no statistically significant difference in the receipt of tocilizumab, as this immunomodulator is used less commonly (approximately 10% in both groups) in local practice. While we did not systematically solicit for or collect adverse effect data in this study, no adverse effects attributable to sotrovimab in the treatment group were identified during clinical chart review.

**Figure 2:**
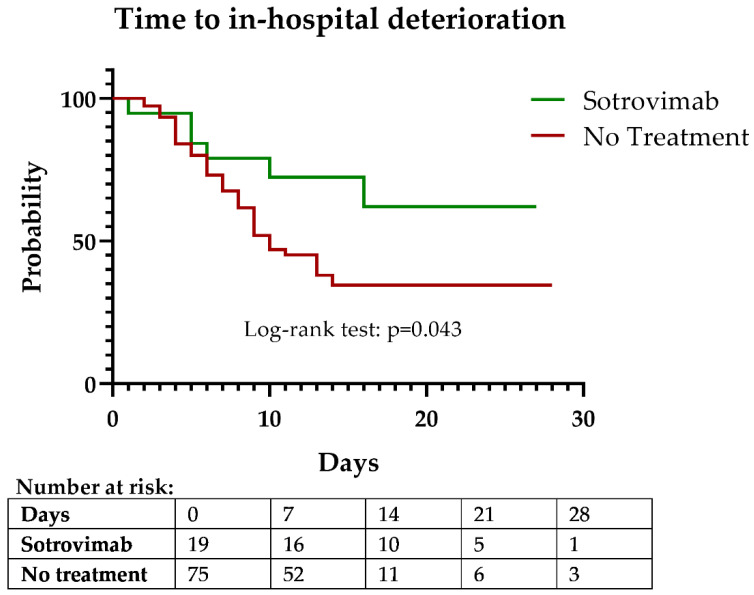
Kaplan–Meier curve of time to in-hospital deterioration (composite outcome of O_2_ requirement, ICU admission, or death).

The Kaplan–Meier curves for time to in-hospital deterioration showed a clear difference between both groups (log-rank test, *p* = 0.043) (Figure 2). Cox proportional hazards model for in-hospital deterioration showed that sotrovimab treatment was protective (hazard ratio (HR), 0.42; 95% confidence interval (CI), 0.18–0.99, *p* = 0.049) in the univariate analysis (Table 2). In a multivariate model, after adjustment for the ISARIC deterioration score, which accounted for differences in age, risk factors, and baseline clinical severity, sotrovimab treatment remained protective (HR, 0.41; 95% CI, 0.17–0.99; *p* = 0.047).

## 4. Discussion

In this cohort of high-risk COVID-19 hospitalized patients, early treatment with sotrovimab within the first five days of illness was associated with a significantly decreased risk of in-hospital deterioration as defined by a composite outcome of oxygen requirement, ICU admission, or mortality. This was despite the fact that patients in the sotrovimab treatment group were older, had more comorbidities, and had a higher baseline ISARIC 4C mortality score, all factors which would have otherwise predisposed them to a less favorable disease course. There was a lower frequency of individual severe outcomes in the univariate analysis comparing treatment and non-treatment groups, although this did not meet statistical significance and may be due to the small sample size of our study.

The rates of progression in our cohort were higher than in phase II/III trials of sotrovimab and REGEN-COV [2,3], which only evaluated mAb treatment in high-risk outpatients with COVID-19, and found relative risk reduction of 85% and 71.3% with sotrovimab and REGEN-COV, respectively. However, there are limited data evaluating the use of sotrovimab in a hospitalized setting. A randomized controlled trial comparing sotrovimab, BRII-196 plus BRII-198 (a separate mAb combination), and a placebo failed to demonstrate improved clinical outcomes with either sotrovimab or BRII-196 plus BRII-198 in hospitalized patients [9]. This may have been due to the late presentation of study participants (inclusion criteria allowed for symptoms up to 12 days, and median day of illness was 8 days for all three groups), underscoring the importance of early identification and prompt treatment of COVID-19 patients with mAb therapy within a narrow therapeutic window. Subgroup analyses, while underpowered, showed differential outcomes based on baseline antibody status, with seronegative patients appearing to benefit more from mAb therapy. Taken together with similar findings from the RECOVERY trial demonstrating mortality reduction with REGEN-COV in baseline seronegative patients [6], this suggests that careful selection of suitable seronegative patients for mAb therapy is required, and that the therapeutic use of sotrovimab in hospitalized patients needs further study. At the time of writing, there are no other prospective clinical trials or large retrospective studies that have evaluated sotrovimab use in hospitalized patients. Our findings provide early data to support the use of sotrovimab in carefully selected high-risk hospitalized patients early in the disease course, where mAbs may potentially have the greatest impact. Patients may be hospitalized for reasons not directly linked to COVID-19 (e.g., for isolation or social reasons, or for other non-COVID-19 medical problems), and such patients may benefit from early mAb treatment to reduce the likelihood of subsequent deterioration and adverse outcomes.

There are several limitations to our study. Firstly, the sample size of the treatment group was small. This was in part due to initial limited availability of mAb treatment, necessitating careful limitations on their use. The mAbs were in general short supply during the study period, and, hence, mAb treatment was carefully triaged and selected for. We also restricted our analysis only to sotrovimab, due to the small number of patients who were treated with REGEN-COV, supplies of which arrived in Singapore after sotrovimab. Nonetheless, we found a statistically significant protective effect with sotrovimab treatment despite this small sample size. Larger studies should be carried out to confirm the utility of sotrovimab, as well as evaluate other mAbs, in similar patient cohorts.

Secondly, decisions regarding sotrovimab treatment initiation were made by individual managing physicians, albeit based on national treatment recommendations, which included, during the study period recommended, consideration of mAbs in persons with an ISARIC 4C mortality score of >9 who were unvaccinated or who were anticipated to have a poor or waned response to vaccination as evidence by serologic testing. Although we adjusted for the baseline ISARIC deterioration score in the multivariable Cox regression model, which somewhat accounted for differences in baseline risk, variation in individual practice may have introduced unrecognized confounding and bias. There were some other baseline differences in baseline characteristics, notably that patients in the treatment group presented earlier than the untreated group (median day of illness 2 vs. 3 days), which may be a result of physicians prioritizing sotrovimab treatment for patients earliest in their treatment course given its mechanism of action and best supportive evidence in this time period [2,3]. However, this difference in day of presentation is unlikely to have significantly impacted or biased the outcome, given the small difference of one day and the fact that we restricted the analysis to only patients presenting within the first five days of illness (i.e., all patients in this study had early presentation).

Lastly, the predominant circulating variant of concern (VOC) in Singapore during the study period was the Delta variant, accounting for almost 100% of all cases [10]. This study was conducted before the arrival of the Omicron variant, and it may, thus, not be generalizable to patients infected with this variant. The Omicron variant is associated with milder disease and lower viral loads [11,12], especially in vaccinated individuals, and thus the role and benefit of pre-emptive treatment with mAbs needs to be further studied. Sub-variants, such as BA.2, may also exhibit diminished in vitro neutralization to sotrovimab [13], although whether this translates to reduced clinical efficacy will need to be further studied in clinical settings.

Further questions remain regarding the optimal use of mAbs. We excluded vaccinated patients in this study, as the utility of mAbs in individuals with pre-existing humoral immunity is less clear. Although US CDC guidelines recommend mAb therapy in high-risk outpatients regardless of prior vaccination status, trials evaluating sotrovimab and REGEN-COV in outpatients excluded previously vaccinated individuals [2,3]. However, antibody levels and vaccine efficacy wane over time, especially after six months [14,15], and mAb therapy may be potentially beneficial in selected vaccinated patients, guided by serologic testing. Retrospective studies have found similar utility of mAbs in high-risk vaccinated patients [16,17], which should be confirmed in larger prospective studies. This will become of increasing relevance as vaccination rates continue to increase, and risk scores or algorithms may be required to identify the best candidates who will benefit the most from pre-emptive mAb therapy. Finally, there is also limited head-to-head comparison of different mAbs, which will increasingly become relevant given the multitude of new mAbs in various stages of development [18]. Head-to-head clinical trials comparing different mAbs, with either early pharmaco-metric or later clinical endpoints, will be useful to establish their different therapeutic uses and the most appropriate target patient population for each mAb and guide therapeutic decision-making.

## 5. Conclusions

Our findings support the use of sotrovimab for early treatment in hospitalized patients with mild-to-moderate COVID-19 at high-risk of disease progression. Further study should characterize patient and disease factors to identify subgroups who will best benefit from early mAb therapy, as well as continually reassess mAb clinical efficacy in the face of evolving SARS-CoV-2 variants and sub-variants.

## Figures and Tables

**Table 1:** Baseline characteristics of study cohort.

	Sotrovimab Group (*n* = 19)	Untreated Group (*n* = 75)	*p*-Value
Demographics and comorbidities			
Female sex	5 (26.3%)	30 (40.0%)	0.30
Age	81 (75–88)	70 (59–80)	0.0023
Number of comorbidities	3 (2–4)	2 (0–3)	0.014
Charlson’s score	1 (1–2)	1 (0–2)	0.057
Diabetes mellitus	7 (36.8%)	23 (30.7%)	0.59
Hypertension	14 (73.7%)	32 (42.7%)	0.021
Hyperlipidemia	9 (47.3%)	26 (34.7%)	0.43
BMI, kg/m^2^	22.0 (18.4–23.8)	21.2 (19.5–25.4)	0.51
Presenting symptoms			
Day of illness, presentation	2 (1–3)	3 (2–5)	0.026
Asymptomatic	2 (10.5%)	6 (8.0%)	0.66
Upper respiratory tract symptoms ^a^	12 (63.2%)	42 (56.0%)	0.61
Fever	9 (47.4%)	44 (58.7%)	0.44
Chest pain	1 (5.3%)	4 (5.3%)	>0.99
Shortness of breath	4 (21.1%)	10 (13.3%)	0.47
Investigations on admission			
WBC count, ×10^9^/L	4.7 (2.9–7.0)	5.7 (4.0–7.9)	0.16
Lymphocyte count, ×10^9^/L	0.73 (0.50–1.14)	0.78 (0.53–1.28)	0.65
Neutrophil count, ×10^9^/L	2.97 (2.09–5.19)	4.02 (2.35–5.65)	0.29
Hemoglobin, g/dL	11.1 (9.9–12.1)	13.2 (11.8–14.3)	0.0001
Platelet count, ×10^9^/L	128 (100–164)	176 (129–248)	0.0028
Serum sodium, mmol/L	136 (131–138)	135 (132–138)	0.94
Serum potassium, mmol/L	3.7 (3.4–3.9)	3.6 (3.2–4.0)	0.79
Serum creatinine, umol/L	100 (84–184)	79 (61–109)	0.0086
Serum urea, mmol/L	7.1 (5.2–9.7)	5.15 (3.5–8.7)	0.020
Serum ALT, U/L	23 (15–32)	24 (15–36)	0.67
Serum AST, U/L	36 (26–59)	40 (26–60)	0.88
C-reactive protein, mg/L	15.9 (7.7–50.4)	31.3 (13–84.2)	0.072
Lactate dehydrogenase, U/L	486 (424–583)	536 (431–704)	0.16
Pneumonia on CXR	7 (36.8%)	30 (40.0%)	>0.99
Parameters on admission			
Temperature, °C	38.0 (36.7–38.4)	37.8 (36.9–38.5)	0.57
Heart rate, bpm	81 (73–92)	89 (77–100)	0.18
Systolic blood pressure, mmHg	141 (130–157)	137 (120–153)	0.45
Diastolic blood pressure, mmHg	69 (59–77)	73 (66–79)	0.13
SpO_2_ at ambient air, %	98 (98–99)	97 (96–98)	0.045
Glasgow Coma Scale < 15	3 (15.8%)	12 (16.0%)	>0.99
ISARIC scores			
ISARIC mortality score	9 (9–11)	7 (4–10)	0.018
ISARIC deterioration score	319 (260–379)	287 (218–372)	0.28
Other treatments (after deterioration)			
Dexamethasone	6 (31.6%)	41 (54.7%)	0.12
Remdesivir	7 (36.8%)	53 (70.7%)	0.008
Tocilizumab	2 (10.5%)	7 (9.3%)	>0.99
Baricitinib	0 (0.0%)	6 (8.0%)	0.34
Clinical outcomes			
O_2_ requirement	6 (31.6%)	41 (54.7%)	0.12
ICU admission	2 (10.5%)	18 (24.0%)	0.35
Mortality	1 (5.3%)	10 (13.3%)	0.45

BMI = body mass index; WBC = white blood cell; ALT = alanine aminotransferase; AST = aspartate aminotransferase; CXR = chest X-ray; SpO_2_ = oxygen saturation; O_2_ = oxygen; ISARIC = International Severe Acute Respiratory and Emerging Infection Consortium; ICU = intensive care unit. Numbers reflected as number (percentage) for categorical variables, and median (interquartile range) for continuous variables unless, otherwise stated. ^a^ Upper respiratory tract symptoms defined as cough, rhinorrhea, sore throat, or anosmia.

**Table 2:** Cox proportional hazards model for in-hospital deterioration.

	Univariate Analysis		Multivariate Analysis	
Variable	Hazard Ratio (95% CI)	*p*-Value	Hazard Ratio (95% CI)	*p*-Value
Sotrovimab treatment	0.42 (0.18–0.99)	0.049	0.41 (0.17–0.99)	0.047
ISARIC deterioration score				
≤250	Ref	-	Ref	-
250–400	1.20 (0.60–2.41)	0.60	1.42 (0.70–2.86)	0.33
>400	2.41 (1.06–5.46)	0.036	2.47 (1.09–5.60)	0.031

Efron’s method was used to handle tied failures, and the proportional hazards assumption was tested by using Schoenfeld’s residuals. CI = confidence interval; ISARIC = International Severe Acute Respiratory and Emerging Infections Consortium; Ref = referent.

## Data Availability

The data presented in this study are available upon request from the corresponding author.
